# The Role of Steroid Hormones in Breast and Effects on Cancer Stem Cells

**DOI:** 10.1007/s40778-018-0114-z

**Published:** 2018-03-13

**Authors:** Denis G. Alferez, Bruno M. Simões, Sacha J. Howell, Robert B. Clarke

**Affiliations:** 1 Breast Biology Group, Division of Cancer Sciences, School of Medical Sciences, Faculty of Biology, Medicine, and Health, Manchester Cancer Research Centre, Wilmslow Road, Manchester, M20 4GJ UK; 2 Department of Medical Oncology, The University of Manchester, The Christie NHS Foundation Trust, Manchester, M20 4BX UK

**Keywords:** Progenitor, Biomarker, Signal pathway, Therapy resistance, Breast cancer stem cells

## Abstract

**Purpose of Review:**

This review will discuss how the steroid hormones, estrogen and progesterone, as well as treatments that target steroid receptors, can regulate cancer stem cell (CSC) activity. The CSC theory proposes a hierarchical organization in tumors where at its apex lies a subpopulation of cancer cells endowed with self-renewal and differentiation capacity.

**Recent Findings:**

In breast cancer (BC), CSCs have been suggested to play a key role in tumor maintenance, disease progression, and the formation of metastases. In preclinical models of BC, only a few CSCs are required sustain tumor re-growth, especially after conventional anti-endocrine treatments. CSCs include therapy-resistant clones that survive standard of care treatments like chemotherapy, irradiation, and hormonal therapy.

**Summary:**

The relevance of hormones for both normal mammary gland and BC development is well described, but it was only recently that the activities of hormones on CSCs have been investigated, opening new directions for future BC treatments and CSCs.

## Introduction

The cancer stem cell (CSC) concept proposes a hierarchical organization of the cells within a tumor, where only a small subset of cells, the CSCs, drives and sustains tumor growth. In preclinical studies using breast cancer models, CSCs are defined as self-renewing cells that can propagate the tumor, which makes them very important in the processes of tumor recurrence, metastasis, and resistance to therapy. These roles make them an important therapeutic target [1].

The first report providing evidence for the presence of breast CSCs (BCSCs) observed that CD44^+^/CD24^low^/ESA^+^/lineage^−^ (named CD44^+^/CD24^−/lo^ henceforth) cells (Table 1), isolated from human breast tumors by fluorescence activated cell sorting (FACS), were enriched for CSCs that were adequate to seed tumors in immune-deficient mice [14]. CD44^+^/CD24^−/lo^ cells were serially passaged and gave rise to tumors containing both CSCs (CD44^+^/CD24^−/lo^) and non-CSCs, suggesting self-renewal and differentiation, respectively. Breast cancers with high CD44 and low CD24 have been associated with the triple negative subtype (negative for estrogen receptor (ER), progesterone receptor (PR), and HER2 receptor) and with poorer prognosis [15, 16].

**Table 1:** Markers of breast cancer stem cells

Markers and reference		Epitope function	Expression in preclinical models	Expression in cancer subtypes	Essays used to evaluate activity
CD44^+^/CD24^−/low^/EpCAM^+^	[2]	CD44 is a ubiquitously expressed receptor for hyaluronan and exerts control over cell growth, migration, and tumor progression.	Not detected in MCF7, T47D, ZR75, SKBR3, and MDA-MB-468	Significantly associated with basal-like and luminal B subtypes, but the inverse associated with luminal A [3].	In vitro proliferation, migration, invasion, colony formation. In vivo tumor formation studies
CD44^+/^CD24^−/low^/EpCAM^+^/Lin^−^					
		CD24, also known as heat stable antigen (HSA), a sialoprotein that is expressed on B cells, T cells, keratinocytes, and myofiber synaptic nuclei and is upregulated in a wide variety of cancers.	Highly expressed in MDA-MB-231, MDA-MB-361, HCC1937		
CD44^+^/CD24^−/low^/Lin^−^					
ALDH1^+^ ALDH1^+^/CD44^+^/CD24^−^	[4]	Aldehyde dehydrogenases (ALDHs) detoxify aldehydes by oxidizing them to carboxylic acids. ALDH1A1 is a cytosolic enzyme that preferentially oxidizes retinaldehyde to retinoic acid.	Highly expressed in MDA-MB-468, MDA-MB-231, HCC1937, SKBR3, MCF7, ZR75 [5].	Significantly associated with HER2+ and basal-Like BC, but negative associated with luminal A [3].	In vivo tumor formation studies.
			Not detected in T47D, MDA-MB-361 [5]. Detected in BT-20, MDA-MB-157, and MDA-MB-231 [6].		
CD133^+^	[7••, 8, 9]	CD133 (also known as prominin 1) is a plasma membrane protein known to be expressed on neural stem cells and hematopoietic stem cells.CD133 high cells may predict for refractory metastatic disease following neoadjuvant endocrine therapy. Associated with higher self-renewal potential and vascular mimicry.	Highly expressed in MDA-MB-468. Not detected in MCF-7, T47D, ZR75, SKBR3, MDA-MB-231, MDA-MB-361, and HCC1937 [5].	Significantly associated staining in Triple negative (71%) [9]. Low expression (staining) in Her2+ (38%) and Luminal (26%) tumors [9].	In vivo tumor formation studies Mediating metastatic progression.
			Not detected in: BT-20 and MDA-MB-157 [6].		
			Detected in MCF-7 ER-low, MCF-7 + Fulv, ZR75 + Fulv and in a Resistant PDX-TamR [7••]. Associated with CSC in Brca^1Δ^11p53^+/−^ mammary tumors		
CD24^+^/CD29^high^	[10]	Integrin beta-1/CD29: A membrane receptors involved in cell adhesion and recognition in a variety of processes including embryogenesis, hemostasis, tissue repair, immune response, and metastatic diffusion of tumor cells	MMTV-wnt (Balb-C) mice	NA	Tumor formation studies
CD24^+^/CD29^−/low^/CD61^+^		Integrin beta-3 (β3)/CD61: integral cell-surface proteins known to participate in cell adhesion as well as cell-surface-mediated signaling.	CSC population in MMTV-wnt (Balb-C) tumors	NA	
			Half of the CSC population in BALB/c-p53^+/−^		
CD24^+^/CD29^+^/CD49f^+^	[11, 12]	Integrin alpha-6/CD49f: a cell surface proteins integral cell-surface involved in cell adhesion as well as cell-surface mediated signaling. CD49f has novel and dynamic roles in regulating the differentiation potential of hMSCs and maintaining pluripotency	CSC population in Brca1-mutant primary mammary tumors(Balb-C)	NA	Tumor formation, migration, and metastasis studies
CD24^high^/CD49f^high^/DNER^high^	[13]	DNER: Delta and Notch-like epidermal growth factor-related receptor		Epithelial cells from reductive mammoplasties.	Colony formation Sphere-forming study In vivo tumor formation studies
CD24^high^/CD49f^high^/DLL1^high^					
		DLL1: a member of the delta/serrate/jagged family involved in cell-to-cell communication			
CD49f^+^/DLL1^high^/DNER^high^					

Basal-like cell lines including MDA-MB-468, MDA-MB-231, and HCC1937; luminal-like cell lines such as T47D, MCF-7, ZR-75, and SKBR-3

*EpCAM* epithelial cell adhesion molecule, *ALDH* aldehyde dehydrogenase, *Fulv* fulvestrant, *BC* breast cancer, *CSC* cancer stem cell

Other strategies have also been used to identify BCSC enriched populations. Mammosphere formation, high aldehyde dehydrogenase (ALDH) activity, and CD49f or CD133 expression are properties that have been utilized to isolate CSCs (Table 1). The mammosphere colony assay tests the capacity of BCSCs to survive in non-adherent culture conditions and to form spherical colonies, called mammospheres [17–19]. The activity of ALDH1, which retinaldehyde to retinoic acid, is detected by an enzymatic assay (ALDEFLUOR) and flow cytometry [20]. The proportion of cells with ALDH1 expression in breast cancer has been shown to correlate with poor prognosis [20–22]. Finally, CD49f and CD133 (Table 1) have recently been shown to enrich for CSCs in chemotherapy resistant triple negative and endocrine-resistant breast cancer, respectively [7••, 23]. The establishment of BCSC markers suitable for all tumors is hindered by intra-tumor and inter-tumor heterogeneity of CSC populations.

At the present time, the most robust enrichment for BCSCs is achieved through the use of CD44^+^/CD24^−/lo^ and ALDH+. These two cell populations have been demonstrated to mark BCSCs in different states and with gene expression resembling either mesenchymal (CD44^+^/CD24^−/lo^ cells) or epithelial characteristics (ALDH+ cells) [24]. A small overlapping population of cells which is both CD44^+^/CD24^−/lo^ and ALDH+ was identified, which suggested that BCSCs possess cellular plasticity and can dynamically switch between mesenchymal and epithelial states. The epithelial–mesenchymal transition and vice-versa (mesenchymal–epithelial transition) can be driven by the tumor microenvironment, with hypoxia or transforming growth factor beta playing key roles in this [25, 26]. It is likely that other signaling factors that have been reported to regulate BCSC activity, such as hormones, will influence this. Herein, we discuss the regulation of BCSC function by the steroid hormones, particularly estrogen and progesterone, and their antagonists [22].

## Estrogen and BCSCs

Estrogen promotes mammary epithelial cell proliferation and is therefore critical for normal breast development, but it also stimulates breast tumor growth through the estrogen receptor (ER) [27]. Estrogen binds to its receptors, ERα and ERβ, which are nuclear ligand-activated transcription factors, to modulate the transcription of target genes [28]. The effects of estrogen in the breast epithelium are mainly mediated by ERα, which has a higher affinity to 17β-estradiol, the physiological form of estrogen, than does ERβ [29]. Transcription factors need nuclear receptor co-regulators to mediate their action on target DNA sequences; in this case, ER signaling is dependent on FOXA1 expression, which promotes local DNA unwinding facilitating the access of ER to DNA [30].

Around three out of four breast tumors express ERα. Its expression is associated with luminal differentiation markers and with a more favorable breast cancer prognosis and is the most important breast cancer predictive factor for endocrine responsiveness [31, 32]. Exposure to high levels of estrogen during women’s lifetime is established to be associated with increased risk of postmenopausal breast cancer [33]. However, exogenous estrogen used as hormone replacement therapy may reduce the risk of invasive breast cancer and breast cancer-specific mortality in postmenopausal women [34]. This paradoxal effect of the role of estrogen in breast cancer initiation and progression might be explained by the different impacts of estrogen on different breast cancer cell types. On one hand, the pro-proliferative function of estrogen in ERα-positive breast cancer cells has been well characterized, but on the other hand, literature detailing the effects of estrogen on breast cancer stem cell (BCSCs) remains relatively scarce [35].

These effects are proposed to occur indirectly via paracrine mechanisms since BCSCs (CD44^+^ CD24^−/lo^ and ALDH^+^ cells) are mostly ERα-negative [36–38]. It has been reported that treatment of CSC-enriched mammosphere population with estrogen decreases the proportion of BCSCs in ERα-positive breast cancer cells as a result of downregulation of embryonic stem cell genes [39]. This observation could in theory explain the better prognosis of ERα-positive tumors [40]. But it also has been shown that ERα-positive breast cancer cells can secrete FGFR and EGFR ligands in response to estrogen, which can act as paracrine mediators to promote CSC activity and expand the fraction of CD44^+^ CD24^−/lo^ cells [41, 42]. In contrast, Axlund et al. reported that estrogen does not change cancer stem/progenitor cell properties on its own [43]. The reasons why some data show a protective effect of estrogen whereas others show that it can enhance cancer cell growth are not yet clear, but likely are related to other underlying differences in the tumor models used in the studies.

More recently, despite the fact that BCSCs do not express the classical ERα, estrogens have been suggested to act directly on BCSCs through the ERα36 variant and ERβ. ERα36 variant activates mitogenic signaling via the AKT/GSK3β pathway and is essential for the maintenance of CD44^+^ CD24^−/lo^ cells of two ER-positive breast cancer cell lines [44]. Ma and colleagues identified ERβ expression to be associated with stem cell markers CD44 and ALDH1 and also to be important for mammospheres formation [45]. Interestingly, ERβ gene expression has been reported to be upregulated in FACS sorted human breast stem cells [46••] compared to the total tumor cell population.

The complex implications of estrogen signaling in human breast cancer cells with stem-like characteristics indicate that further studies are needed to fully elucidate the effects of estrogens on BCSCs. Standardization of experimental conditions is warranted since the use of different BCSC markers, models, or culture conditions alters the analysis of CSC activity.

## Anti-estrogen Drugs and BCSCs

Anti-estrogen therapies are used for breast cancer treatment of ER-positive tumors in both the adjuvant and metastatic settings. The principal classes of drugs are selective estrogen receptor modulators (SERMs, e.g., tamoxifen) and downregulators (SERDs, e.g., fulvestrant) as well as aromatase inhibitors, that reduce estrogen synthesis [47]. Since BCSCs are mostly ER-negative, they are not targeted by anti-estrogen therapies and several publications have reported that these therapies enrich for cells with BCSC characteristics.

Tumors treated with letrozole (aromatase inhibitor) increased in CD44^+^CD24^−/lo^ mammosphere forming cells [48]. Piva and colleagues reported that tamoxifen-resistant MCF-7 cells have increased CD44^+^CD24^−/lo^ and ALDH+ populations and form more mammospheres than the parental cells. In addition, they established that expression of the embryonic stem cell marker SOX2 and consequent activation of WNT signaling pathway was key for BCSCs survival after tamoxifen treatment [49]. Another study showed that ERα36 promotes tamoxifen resistance by increasing the proportion of CD44^+^CD24^−/lo^ cells and mammosphere-forming cells [44]. Our group has shown that BCSCs (ALDH+ cells) are enriched following anti-estrogen treatment of breast cancer cells both in vitro using patient samples and in vivo using patient-derived xenografts. We also found that ALDH+ cells have high expression of JAG1 ligand and NOTCH4 receptor and that high ALDH1 expression predicts anti-estrogen resistance in women treated with tamoxifen [38]. Recently, two different studies from Sansone and colleagues demonstrated how the transfer of miR-221 or full mitochondrial DNA from cancer-associated fibroblasts to breast cancer cells through circulating extracellular vesicles could promote an exit from dormancy of BCSCs (CD133+) leading to endocrine therapy resistance [50, 51].

Together, these findings suggest that inhibition of estrogen signaling in breast cancer cells may lead to an increase of the proportion of BCSCs. It still needs to be addressed whether this phenomenon occurs through selection and survival of the ER negative BCSCs, through induction of BCSCs characteristics in the ER+ cells, or by both processes. Either way, we hypothesize that BCSCs that survive anti-estrogen treatments can enter a dormant state and eventually re-initiate tumor growth, sometimes several years after the therapy. Further interest in this field has given rise to several clinical trials directly targeting CSCs (via recognized markers like ALDH) or using different signaling pathways linked to CSCs (Table 2).

**Table 2:** Clinical trials with CSC targeting therapies or with a CSC rationale/endpoint

NCT number	Compound agent	Mode of action	Recruitment	Disease	Combined treatment	Outcome measure endpoints	Phases	Study type
CSC targeting pathways								
NCT02157051	STEMVAC	Multiepitope vaccine CD105/Yb-1/SOX2/CDH3/MDM2	Recruiting	Advanced breast cancer	NA	Immunologic efficacy by increase in Th1 cell immunity	1	Interventional
NCT02063893	Cytotoxic T cells	ALDH^high/low^	Completed	Metastatic breast cancer	NA	Safety of immunization and immune responses due to vaccine	1 & 2	Observational
NCT02254005	Bivatuzumab mertansine	Antibody against CD44v6 conjugated with chemo	Completed	Advanced breast cancer	NA	DLT and MTD	1	Interventional
NCT02254031		CSC	Terminated	Metastatic breast cancer	NA	DLT and MTD	1	Interventional
NCT00001504	9-*cis*-retinoic acid		Completed	Breast neoplasms to breast cancer	NA	DLT and MTD of combination	1	Interventional
NCT01281163	Lapatinib		Terminated	ER, HER-positive advanced breast cancer	MK2206	MTD of combination, monitor BCSC biomarkers	1	Interventional
NCT00949013			Completed	Breast cancer	NA	Correlation of ALDH1 with disease-free survival and overall survival	NP	Observational
NCT00923052			Terminated	Breast cancer	NA	To characterize quantitatively and qualitatively CSC in solid tumors	NP	Observational
NCT01424865	Trastuzumab		Unknown status	Breast cancer	NA	ALDH1 expression and association with outcomes regardless of HER2 staining	NP	Observational
NCT01641003			Unknown status	Breast cancer	NA	Breast CSC percentage	NP	Observational
Notch targeting pathways								
NCT00756717	MK-0752	γ-Secretase inhibitor	Active	Breast cancer	NA	MTD of MK-0752 in the presurgical setting	1 and 2	Interventional
NCT00106145			Completed	Advanced breast cancer	NA	DLT and MTD	1	Interventional
NCT00645333			Completed	Metastatic breast cancer	Docetaxel, pegfilgrastim	DLT and MTD	1 and 2	Interventional
NCT01149356	RO4929097		Terminated	ER-positive advanced breast cancer	Exemestane, goserelin acetate	Time to relapse and overall survival	1	Interventional
NCT01151449			Terminated	Triple negative breast cancer	NA	Overall response rate using RECIST and overall survival	2	Interventional
NCT01208441			Terminated	ER-positive advanced breast cancer	NA	DLT and MTD	1	Interventional
NCT01238133			Terminated	Triple negative breast cancer	Paclitaxel, carboplatin	DLT and MTD of combination	1	Interventional
NCT02299635	PF-03084014		Terminated	Triple negative breast cancer	NA	ORR and PFS	2	Interventional
NCT01876251			Terminated	Metastatic breast cancer	Docetaxel	DLT and MTD	1	Interventional
NCT02298387	Navicixizumab (OMP-305B83)	Anti-DLL4/VEGF bispecific	Active	Advanced solid tumor	NA	DLT and MTD	1	Interventional
Wnt targeting pathways								
NCT01973309	OMP-18R5	Anti-frizzled 7	Active	Metastatic breast cancer	Paclitaxel	DLT and MTD of combination	1	Interventional
NCT01608867	OMP-54F28	Frizzled-8 receptor and a human IgG1Fc fragment.	Completed	Advanced solid tumor	NA	DLT and MTD of combination	1	Interventional
NCT01431872		Measuring DKK1, WNT signaling inhibitor	Completed	Breast cancer	NA	Monitoring estrogen levels which suppresses DKK1	NP	Observational
Hedgehog signaling targeting pathways								
NCT02694224	Vismodegib	Smoothened receptor (SMO)	Recruiting	Breast cancer	Paclitaxel, epirubicin, cyclophosphamide	DLT and MTD of combination	2	Interventional
NCT01071564			Terminated	Triple negative breast cancer	NA	DLT and MTD	1	Interventional
Microenvironment targeting pathways								
NCT02001974	Reparixin	CXCR inhibitor	Completed	Metastatic breast cancer	Paclitaxel	Pk profile of orally administered reparixin and BORR, among outcomes: expression of ALDH1 and CD44 on tumor biopsies	1	Interventional
NCT02370238			Recruiting	Metastatic breast cancer	Paclitaxel	PFS and ORR	2	Interventional
NCT01861054			Terminated	Breast cancer	NA	Characterization of markers of CSCs	2	Interventional

*BCSC* cancer stem cell, *DLT* dose limiting toxicity, *MTD* maximum tolerated dose, *PFS* progression-free survival, *ORR* objective response rate, *CBR* clinical benefit rate, *BORR* best overall response rate

### Progesterone and BCSCs

Progesterone plays a pivotal role in lobuloalveolar development of the mouse mammary gland during pregnancy [52, 53]. In premenopausal women, breast epithelial cell proliferation is highest in the luteal phase of the menstrual cycle during maximum progesterone secretion [54, 55]. Progesterone signaling is mediated by the progesterone receptor (PR), expressed as two isoforms (PRA and PRB) that are only different by a third activation domain on the 5′ end of PRB [56]. Importantly, the ratio of these two isoforms is key in the normal development of the mammary gland [57]. Further evidence from isoform-specific murine mutants demonstrates that mammary gland morphogenesis is linked to PRB, whereas PRA plays a prominent role in the ovarian homeostasis [58, 59]. The gene expression patterns while largely overlapping indicate that PRB can regulate gene expression of more genes in comparison to its counterparts [60].

In normal human breast cells, stimulation with progesterone in matrix-embedded culture increases bi-potent cell numbers [61]. Evidence in mouse models corroborates that progesterone and PR signaling drives mammary gland development by expansion of the mammary stem cell population; this signaling is also appropriated in carcinogen-induced mammary tumor formation [62–64]. In established cancer cell lines, progestin administration leads to an increase of progenitor cells and CSC markers [65].

In the normal breast epithelium, the ratio of the PR isoforms remains balanced, but this is disrupted in the cancer setting, favoring the expression of PRA [61, 66]. The increased risk of developing breast cancer has been linked to atypical hyperplasia [67] which often exhibits loss of PRB, equally, altered ER expression, sole PRA expression, and preferential PRB loss is also reported in the normal breast tissue of women with germline BRCA1/2 mutations [68]. Such women demonstrate PR isoform imbalance and double the circulating progesterone levels compared to matched controls; however, the cause and significance of these findings remain obscure [69].

During mammary gland expansion, PR mediates proliferation via paracrine signals, including RANKL (receptor activator of nuclear factor-κB ligand) and Wnt4. These signals are secreted from PR+ sensor cells and act on PR− progenitor cells, expressing RANK and the Wnt receptors Frizzled and LRP5/6 [63, 64]. In multiple rodent models, deletion or inhibition of PR or the RANK/RANKL pathway results in significant reduction in mammary carcinogenesis [62, 63, 70, 71]. More recently, Nolan and colleagues have also shown the potential of RANKL as a therapeutic target in a Brca1-deficient mouse model, while in normal breast tissue of BRCA1-mutation carriers, identifying luminal RANK(+) progenitors that are highly proliferative and bear a molecular signature similar to that of basal-like BC, this indicates RANKL inhibition as a promising strategy in the prevention setting [72•].

One of the mediators of progesterone-induced stem/progenitor cell functions in normal mammary gland is CXCR4/CXCL12 [73]. Signaling by progesterone occurs in a paracrine manner on luminal cells expressing CXCL12 while CXCR4 expression is also induced in both basal and luminal PR− cells. Inhibition of CXCR4-CXCL12 signaling axis can arrest the progesterone induced expansion of mammary stem/progenitor cells. Ginestier and colleagues translated the inhibition of CXCR1 with either a specific blocking antibody or by methanesulfonamide (a CXCR inhibitor known as Reparixin), in which both depleted the CSC population of two BC cell lines in vitro and in vivo [74]. This approach is currently under evaluation in the clinic, using reparixin in both early and advance breast cancer (Table 2). A further effect of progesterone is the secretion of growth hormone (GH) in human breast epithelial cells, driving proliferation of the stem/progenitor breast cells expressing growth hormone receptor (GHR) [75].

Similarly, the increase in CK5^+^ cell population (linked to tumor-initiating properties and therapy resistance) and CD44^hi^ or CD44^+^CD24^−^ BCSCs has been linked to progesterone in several ER+PR+ cell lines but particularly in T47D cells, which have high PR levels, through gene amplification, even in the absence of estrogen [65, 76–78]. In cell lines where PR expression is still dependent on estrogen, co-stimulation with estrogen and progesterone is required, while estrogen alone was not able to induce BCSCs.

In terms of potential mechanisms, reports have shown that PR signaling inhibits the expression of miR-29 and miR-141, while de-repressing KLF4 and STAT5A, respectively [77, 79]. Both studies showed expansion of the CK5+/CD44+ CSC population with an increase in colony formation and in vivo tumor initiating capacity. KLF4 is a transcription factor required for maintenance of both BCSCs [80] and pluripotency in embryonic stem cells [81] whereas STAT5A is a transcription factor that regulates the mammary luminal progenitor population [82]. The maintenance of leukemic stem cells heavily depends of BCL6 expression while also essential for progesterone-induction of CK5+ cells in luminal breast cancer [83]. This progesterone-induced expression of BCL6 is suppressed by prolactin, further demonstrating the interplay taking place in hormonal signaling in the regulation of BCSCs [84]. The paracrine signaling taking place in the normal mammary gland between PR+ and PR− cells may indeed be acting in the same fashion with PR-BCSCs. Furthermore paracrine signaling of non-endogenous overexpressed RANKL in human breast cell lines increases the CD44+CD24− BCSC pool, promoting tumor initiation and metastasis [85]. However, despite strong preclinical data, clinical trials of denosumab, a monoclonal antibody targeting RANKL, have not translated to any improvement in cancer specific survival despite their valuable role in reducing skeletal complications from bone metastases. Altogether, this large body of evidence indicates that the expansion of both normal and BCSC is largely or in part driven by progesterone, although the exact mechanisms remain to be elucidated. Inhibition of PR directly or its paracrine/downstream mediators could translate to rational drug targets for breast cancer prevention and therapy.

## Anti-progesterone Drugs and BCSCs

The Women’s Health Initiative study reports that combination of estrogen with progestin (synthetic progesterone derivative), but not estrogen alone, was associated with an increased breast cancer incidence and mortality [86]. Tumorigenesis in the mammary gland can be attributed to the effects of progesterone signaling expanding the stem cell pool, which may transform to BCSCs and eventually lead to the formation of ER^+^/PR^+^ tumors [87]. Recent reports in vitro have shown that natural and synthetic progestins can increase CSC-related markers ALDH^high^ and CD44^high^ (Table 1) and that this enrichment of a subpopulation of cancer cells may be of functional significance in the development of BC in vivo [65]. The potential of progestin modulators as anti-tumor agents has recently been addressed using a patient derived xenograft model of breast cancer; investigators showed ulipristal acetate, a selective progesterone receptor modulator translated to significant anti-tumor effect, with reduction in Ki67 and Cyclin D1 [88].

During much of the 1990s, a great investment in anti-progestins as therapeutic agents was seen. Several trials were initiated for BC and other indications, as monotherapies and or in combinations. However, despite much interest, no anti-progestin is currently used as the recommended standard of care in any cancer setting either through lack of activity or tolerability. An example of these was the onapristone phase I trials which showed liver function test abnormalities, halting its clinical development [89, 90]. Recent years have seen a new series of clinical trials (Table 3) using anti-progestin drugs like mifepristone and onapristone (the latter, now administered in a new formulation to avoid previous observed hepatoxicity) in breast cancer and other solid tumors [91–95]. Based on recent research literature, these drugs may target BCSCs in ER^+^/PR^+^ tumors; although hypothetical, this merits further investigation. Trials in the prevention setting are also investigating the effects of ulipristal acetate, a selective progesterone receptor modulator, assessing proliferation and CSC markers in normal breast tissue [94]. New evidence on the complex interaction of estrogen and progesterone now elucidates the co-regulation of proliferative signaling under both steroid hormones. Progesterone acting through PR is able to ameliorate the effects of estrogen by reducing it activation of downstream effectors [96••]. This may offer and explanation as to why double positive endocrine tumors (ER^+^/PR^+^) are classified as less aggressive than single ER + breast cancers, translating to better prognosis [96••, 97]. A hypothesis currently being evaluated in clinical trials investigates the potential benefits of PR agonists, as single agents in improving and prolonging response or in combination with cholecalciferol or letrozole (Table 3). The renewed interest in PR as potential therapeutic target may hold clinical benefits by either modulating ER co-regulations by progestins or by reducing the progenitor pool via PR signaling. Despite new advances and insights into the effects of PR, more clinical work is need to validate the preclinical data.

**Table 3:** Clinical trials involving progesterone receptor modulation

NCT number	Compound/agent	Mode of action	Recruitment	Disease	Combined treatment	Outcome measures	Phases	Study type
NCT03306472	Megestrol acetate	PR agonist	Recruiting	Breast cancer	Letrozole	Determination PD profile of orally administered megestrol acetate	2	Interventional
NCT01608451	Inj. progesterone	PR agonist	Active	Advanced breast cancer	Cholecalciferol (vit D analogue)	To evaluate PFS and OS	3	Interventional
NCT00123669	Hydroxyprogesterone caproate (OHPC)	PR agonist	Active	Breast neoplasms		To evaluate PFS and OS	2/3	Interventional
NCT02651844	Mifepristone	PR antagonist	Recruiting	Breast cancer		Determination PD profile of orally administered Mifepristone	1/2	Interventional
NCT01138553	Mifepristone	PR antagonist	Terminated	Advanced breast cancer		Determination PD profile of orally administered Mifepristone	1	Interventional
NCT02046421	Mifepristone	PR antagonist	Active	Advanced breast cancer	Carboplatin and gemcitabine hydrochloride	DLT and MTD of combination	1	Interventional
NCT02014337	Mifepristone	PR antagonist	Active	Breast cancer	Eribulin	DLT and MTD of combination	1	Interventional
NCT01493310	Mifepristone	PR antagonist	Active	Advanced breast cancer	Nab-paclitaxel	DLT and MTD of combination	1	Interventional
NCT01800422	Telapristone acetate	Selective progesterone receptor modulator	Active	Breast cancer		Determination PD profile of orally administered telapristone acetate	2	Interventional
NCT02314156	Telapristone acetate	Selective progesterone receptor modulator	Recruiting	BRCA1 mutation carrier breast cancer		Determination PD/PK profile of telapristone acetate	2	Interventional
NCT02052128	Onapristone	Selective progesterone receptor modulator	Unknown status	Breast cancer		Determination PD/PK profile of onapristone	1/2	Interventional
NCT02052128	Onapristone	Selective progesterone receptor modulator	Unknown status	Breast cancer		Determination MTD and PK profile of onapristone	1/2	Interventional
NCT02408770*	Ulipristal acetate	Selective progesterone receptor modulator	Unknown status	Normal breast tissue (breast cancer)		Determination PD profile of ulipristal acetate in normal breast epithelium	2	Interventional
NCT00555919	Lonaprisan	Selective progesterone receptor modulator	Completed	Metastatic breast cancer		To evaluate PFS, ORR and OS	2	Interventional

*DLT* dose limiting toxicity, maximum tolerated dose, *PFS* progression-free survival, *CBR* clinical benefit rate, *BORR* best overall response rate, *ORR* objective response rate, *OS* overall survival. * Trial designed in the prevention setting

## Conclusions

**Fig. 1:**
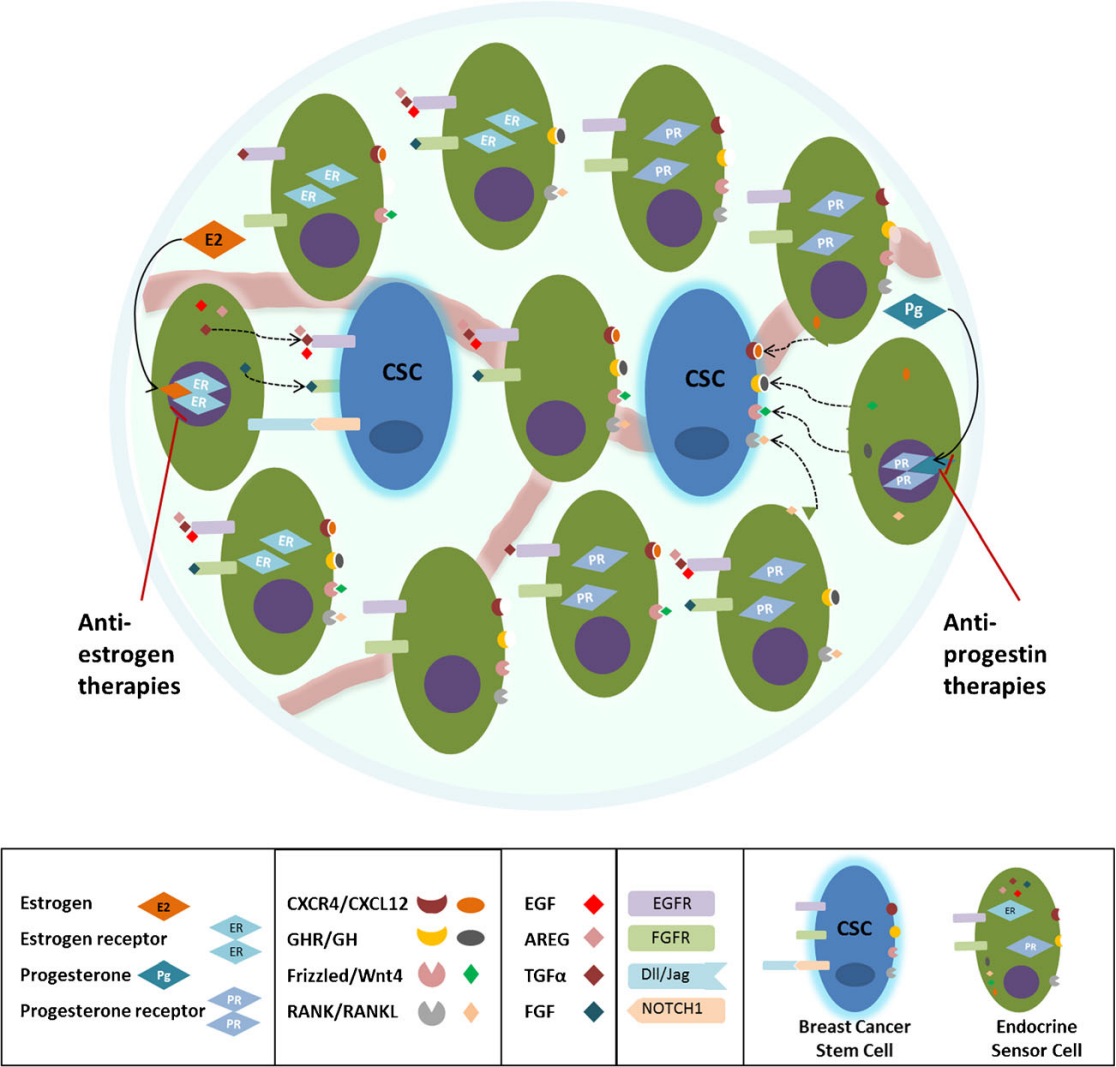
Representation of juxtacrine and paracrine signals involved in estrogen and progesterone regulation of BCSCs. Estrogen (E2) and progesterone (Pg) bind to their receptors along with nuclear transcription factors, respectively, regulating expression of downstream target genes. Estrogen sensor cells (non-BCSCs) increase transcription of EGF (epidermal growth factor), AREG (amphiregulin), TGFα (transforming growth factor α), and FGF (fibroblast growth factor), which will signal to the BCSCs through the EGFR and FGFR receptors. Non-BCSCs can also signal with BCSCs via Notch signaling. Progesterone sensor cells (non-BCSCs) upregulate the transcription of several key signaling factors. Regulation of BCSCs via Pg may occur via activation of RANK/RANKL, Wnt receptors/Wnt4, CXCR4/CXCL12, and GHR/GH paracrine signaling (dashed lines). Estrogen and progesterone-induced signals can be blocked by anti-estrogens (e.g., tamoxifen and fulvestrant) and anti-progesterone drugs (e.g., mifepristone and onapristone)

In breast cancer, both estrogen and progesterone signaling have effects on CSC activity. BCSCs are reported to be low or negative for steroid hormone receptors, and therefore, effects must be indirect, mediated through paracrine or juxtacrine cell–cell signaling (Fig. 1). It remains possible that there is a degree of autocrine signaling downstream of hormones that regulates some BCSCs. The effects of estrogen and progesterone have only been partly described in cancer, for progesterone particularly; there is more data from normal mammary epithelium than from cancer. For estrogen, there is evidence that following in vitro treatment of serum-starved breast cancer cells, CSC activity is upregulated and that this is regulated by EGF, FGF, or Notch1 receptors, indicating indirect, paracrine or juxtacrine signaling between cells (Fig. 1). In contrast, anti-estrogens, such as tamoxifen or fulvestrant, block direct estrogenic effects on cell proliferation, and indirect signals to the ER− BCSCs. Surprisingly, however, tamoxifen can actually increase BCSC activity in mammosphere colony culture [38, 39, 98], and more recently, the same has been confirmed for both tamoxifen and fulvestrant in vivo [38]. In breast cancer, both estrogen and progesterone signaling have effects on CSC activity. BCSCs are reported to be low or negative for steroid hormone receptors, and therefore, effects must be indirect, mediated through paracrine or juxtacrine cell–cell signaling (Fig. 1). It remains possible that there is a degree of autocrine signaling downstream of hormones that regulates some BCSCs. The effects of estrogen and progesterone have only been partly described in cancer, for progesterone particularly; there is more data from normal mammary epithelium than from cancer. For estrogen, there is evidence that following in vitro treatment of serum-starved breast cancer cells, CSC activity is upregulated and that this is regulated by EGF, FG,F or Notch1 receptors, indicating indirect, paracrine or juxtacrine signaling between cells (Fig. 1). In contrast, anti-estrogens, such as tamoxifen or fulvestrant, block direct estrogenic effects on cell proliferation and indirect signals to the ER− BCSCs. Nevertheless, as mentioned previously, tamoxifen can actually increase BCSC activity [38].
